# Predictive value of DWI-FLAIR Mismatch in patients with Ischemic Stroke and receiving Endovascular treatment beyond Time Window

**DOI:** 10.12669/pjms.37.2.3293

**Published:** 2021

**Authors:** Shan Cao, Hui Dong

**Affiliations:** 1 Shan Cao, Telemedicine Center, Baoding No.1 Central Hospital, Baoding, 071000, Hebei, China; 2 Hui Dong, Department of Emergency, Baoding No.1 Central Hospital, Baoding, 071000, Hebei, China

**Keywords:** Beyond time window, DWI-FLAIR mismatch, EVT

## Abstract

**Objective:**

To investigate the efficacy and safety of endovascular treatment in patients having acute ischemic stroke with over-time window under DWI-FLAIR mismatch.

**Methods:**

From January 2018 to January 2020, 80 patients who met the research criteria in the First Central Hospital of Baoding, China were selected. According to the time of onset, they were divided into test group and control group, with 40 cases in each group. Forty patients in the test group were beyond time window (6~24h) and the MRI showed a DWI-FLAIR mismatch. Forty patients in the control group were within the time window (< 6h). All patients received endovascular treatment (EVT). The mRS, NIHSS and infarct volume of patients in the test group were compared and analyzed before and 30 and 90 days after treatment, as well as the indicators of both groups of patients before and after treatment, to determine therapeutic effect in patients receiving EVT beyond time window. Meanwhile, the recanalization of the blood vessel and the incidence of cerebral hemorrhage of patients in both groups were compared to determine the safety in patients receiving EVT beyond time window under DWI-FLAIR mismatch.

**Results:**

The mRS, NIHSS and infarct size in the test group were significantly improved before and 30 and 90 days after treatment (p<0.05). The test group showed no significant difference in mRS, NIHSS and other indicators when compared with the control group (p>0.05). There was no significant difference in the rate of recanalization of the blood vessel and intracranial hemorrhage after treatment between both groups (p>0.05).

**Conclusion:**

DWI-FLAIR mismatch can be used as an objective imaging basis for intravascular interventional therapy in patients with stroke with over-time window and large vessel occlusion. It has the advantages of short examination time, non-invasiveness, no need for contrast agents, simple implementation, clear guidance.

## INTRODUCTION

As people’s living habits are changing, the incidence of hypertension, hyperlipidemia and obesity is increasing, and the ensuing incidence of cardiovascular and cerebrovascular diseases is also on the rise year by year. Ischemic stroke is a cerebrovascular disease often complicated with hypertension and hyperlipidemia, which will bring a higher disability rate and seriously affect the quality of life of patients. Currently, it is believed that for patients with ischemic stroke who also have large vessel occlusion (LVO), thrombolysis in the vessels using recombinant tissue plasminogen activator (rt-pa) within six hour (6h) of onset (within the time window) can achieve better therapeutic effects.^1^ For patients 6h after onset (beyond time window), thrombolysis can also achieve a certain effect if the MRI simultaneously show DWI-FLAIR mismatch.^2^ With the development of endovascular treatment (EVT), the common EVT such as thrombectomy and putting intravascular stent are quite effective in clinical practices, and have become the gold standard in treating patients with ischemic stroke who also have LVO within the time window; while for patients of time window beyond, especially when MRI shows a DWI-FLAIR mismatch, there are still few relevant studies on the clinical effect and prognosis evaluation after EVT. We analyzed the clinical data and prognostic data of 40 patients who underwent EVT with a DWI-FLAIR mismatch over time window. It is reported as follows.

## METHODS

### Ethical approval

The study was approved by the Institutional Ethics Committee of Baoding No.1 Central Hospital (dated: July 17, 2020), and written informed consent was obtained from all participants.

### Patients materials

A total of 80 patients hospitalized from January 2018 to January 2020 were selected, all of whom met the following inclusion criteria:

1. Aged ≥18 years.
2. Had clear focal neurological signs (especially if there was a disturbance of consciousness or gaze, etc.).
3. MRA suggested the acute anterior circulation large artery occlusion (such as the internal carotid artery, proximal of segment M1 or M2 of middle cerebral artery, including tandem lesions).
4. MRI showed DWI-FLAIR mismatch (DFM).
5. Patients actively cooperated during the treatment.
6. All patients signed EVT ICF and the hospital ethics committee approved.

The patients were divided into two groups according to the duration from onset to receiving EVT, including 40 cases in the test group of 6 to 24 hours (beyond time window), and 40 cases in the control group of <6 hours.

All subjects were required to complete the examination:

1. Blood analysis.
2. Blood glucose, blood lipids.
3. ECG examination.
4. Prothrombin time (PT) / international standardized ratio (INR) and activated partial thromboplastin time (APTT).
5. The head MRI sequence included MRA, DWI, FLAIR, etc.

There was no significant difference in general data and clinical biochemical indexes between both groups (Table-I) (p>0.05), which were comparable.

**Table-I:** Evaluation of general data and biochemical indexes of the test group and the control group (X̄ ±S) n=40.

*Index*	*Test group*	*Control group*	*t/Χ^2^*	*P*
Age (years)	71.3±9.7	68.5±7.5	1.44	0.15
Male (cases %)	21 (52.5%)	20 (50%)	0.00	0.37
Hypertension (%)	32 (80%)	27 (67.5%)	1.16	0.11
Diabetes (%)	19 (47.5%)	21 (52.5)	0.00	0.16
***Arrhythmia***				
Atrial fibrillation (%)	2 (5%)	3 (7.5%)	0.21	0.32
Atrial premature beat (%)	3 (7.5%)	0 (0%)	3.12	0.12
PVC (%)	3 (7.5%)	2 (5%)	0.21	0.32
Hyperlipidemia (%)	30 (75%)	32 (80%)	0.29	0.18
Blood WBC ( 10^9^/L)	7.50±0.83	7.35±0.77	0.17	0.83
***Infarct site***				
Internal carotid artery (%)	7 (17.5%)	9 (22.5%)	0.31	0.19
Middle cerebral artery M1 (%)	20 (50%)	17 (42.5%)	0.45	0.14
Middle cerebral artery M2 (%)	13 (32.5%)	14 (35%)	0.06	0.18

P>0.05

### MRI multi-modal sequence examination

All patients were given cranial MRI before EVT using Philips 3.0T MRI system. Multi-modal sequence examinations included: T1, T2, FLAIR, DWI, MRA, 3-dimensional arterial spin label imaging (3DASL) examination. The acquired image was processed using Func-tool software system and jointly analyzed by 2 MRI technicians and a neurologist; then the long diameter and the transverse diameter of the maximum FLAIR and DWI infarction section and the number of infarction layer were measured, and the infarct volume was calculated (long diameter × transverse diameter × number of layers). Meanwhile the condition of large vessels in the lesion area was judged by MRA. Criteria for DWI-FLAIR mismatch (DFM): DFM = (DWI infarct area-FLAIR infarct area) ÷ DWI infarct area × 100%, and < 20% was regarded as DWI-FLAIR mismatch (DFM).^3^

### EVT

All patients underwent the treatment under local infiltration anesthesia combined with intravenous sedation. The patient was staying in a supine position, and the femoral vein under the right groin was usually the puncture point. After successful puncture, a 0.014 inch Floppy micro-guide wire (Stryker Corp, Kalamazoo, MI, USA) was inserted, and then a Rebar (18 or 27) (Covidien, St. Louis, MO, USA) micro-catheter was inserted under the guide wire. Contrast agent was injected to understand the thrombus and distal blood vessels. The Solitaire AB (Covidien, St. Louis, MO, USA) stent was used to enter the thrombus site through the microcatheter, the thrombus was fully embedded in the mesh of the stent and fixed, and the stent was withdrawn from the body together with the microcatheter.

### Assessment of revascularization of the blood vessel

After the patient was treated, the recanalization of the blood vessel was evaluated using cervical vascular Doppler and transcranial Doppler (TCD). The recanalization of the blood vessel was graded by TICI. Grade 2b was defined as successful revascularization, and Grade-3 represented complete perfusion in the arterial distribution area.

### Judgment of efficacy

All patients were evaluated by a professional neurologist using mRS and NIHSS scoring system before treatment, and reassessed 30 days and 90 days after treatment, and recorded the changes in infarct size before and after treatment. The differences between both groups of patients and the differences between the test group before and after treatment were compared to evaluate the efficacy of EVT in the test. Safety assessment: The incidence of symptomatic intracranial hemorrhage (NIHSS increase ≥ 4 scores) after EVT in both groups was analyzed to evaluate the safety of EVT in patients beyond the window time under DFM.

### Statistical method

The data was analyzed using SPSS19.0. Measurement data are expressed as ±S. The data between the two groups were compared by t test of two independent samples. Paired T test was used for comparison between groups before and after treatment. The rate was compared using the χ^2^ test, and the difference was statistically significant when p<0.05.

## RESULTS

The data analysis of the mRS, NIHSS, and infarct volume (see Table-II) of the test group before treatment and 30 and 90 days after treatment indicated that the MRS was significantly improved 30 days after treatment compared with before treatment (p<0.05). However, after 90 days of treatment, the improvement was not obvious compared with that after 30 days of treatment (P>0.05). The NIHSS and infarct volume were improved significantly 30 days after treatment compared with those before treatment (p<0.05), and were further improved after 90 days after treatment. There was a significant difference when compared with that after 30 days of treatment.

**Table-II:** Efficacy comparison of the test group before treatment and 30 days and 90 days after treatment ( X̄ ±S)n=40

*Index*	*Before treatment*	*30 days after treatment*	*90 days after treatment*	*t*			*p*		

				t1	t2	t3	P1	P2	P3
mRS	4.2±0.7	2.2±0.4	1.8±1.1	13.72	11.64	2.02	0.00	0.00	0.05
NIHSS	20.70±2.24	17.23±3.31	7.35±2.24	5.49	20.65	15.63	0.00	0.00	0.00
Infarct volume (mL)	97.21±17.83	14.36±7.47	8.13±2.19	27.11	31.36	5.06	0.00	0.00	0.00

***Note:*** t1p1= Before treatment VS 30d after treatment, t2p2= Before treatment VS 90d after treatment, t3p3=30d after treatment VS 90d after treatment.

The data analysis of the test group and the control group before and after treatment is shown in Table-III. MRS and infarct volume differed 90 days after treatment, of which the control group was lower than that in the test group (p<0.05); there were no significant differences in the other indicators between the 2 groups before and after treatment. It might be because the average age of the control group was relatively young. Another possible reason was that EVT within the time window may have advantages in long-term recovery.

**Table-III:** Data analysis of test group and control group before and after treatment (X̄ ±S ) n=40.

*Index*	*Collection time*	*Test group*	*Control group*	*t*	*p*
mRS	Before treatment	4.2±0.7	4.5±1.3	1.29	0.20
	30d after treatment	2.2±0.4	2.0±0.7	1.57	0.12
	90d* after treatment	1.8±1.1	1.4±0.4	2.16	0.03
NIHSS	Before treatment	20.70±2.24	22.15±5.33	1.59	0.12
	30d after treatment	17.23±3.31	16.20±3.36	1.38	0.17
	90d after treatment	7.35±2.24	8.05±2.07	1.45	0.15
Infarct volume (mL)	Before treatment	97.21±17.83	99.05±16.33	0.48	0.63
	30d after treatment	14.36±7.47	13.20±4.17	0.86	0.39
	90d* after treatment	8.13±2.19	5.10±1.39	7.39	0.00

* p<0.05

After treatment with EVT, both groups of patients achieved TICI grade 2b or grade 3 recanalization, and there was no significant difference of the grade 2b and grade 3 recanalization rates between the two groups (p>0.05). After EVT, there were 5 cases of intracranial hemorrhage in the test group and one case in the control group, with no significant difference (χ^2^=2.88, p=0.09), and the incidence of symptomatic intracranial hemorrhage and asymptomatic intracranial hemorrhage was not statistically different between the two groups (p>0.05) (Table-IV).

**Table-IV:** Analysis of recanalization and intracranial hemorrhage after treatment in the test group and the control group (X̄ ±S) n=40.

*Item*	*Test group*	*Control group*	*Χ^2^*	*p*
***TICI grade***				
Grade 2b	13	8	1.61	0.09
Grade 3	27	32	1.76	0.07
***Intracranial hemorrhage***				
Symptomatic	2	0	2.05	0.25
Asymptomatic	3	1	1.05	0.25
Total	5	1	2.88	0.09

P>0.05

## Discussion

Ischemic stroke is one of the brain strokes, the common symptoms are speech disorder and half of the body weakness,^4^ with a high fatality rate and disability rate. The clinical prognosis of patients with acute ischemic stroke is often poor. Early diagnosis and treatment can prevent the progression of the condition and the progressive exacerbation of nerve damage, so time is the brain.^5^ For patients with acute ischemic stroke, especially for those complicated with LVO, intravenous thrombolysis and EVT are commonly used in clinical practices at present, which can effectively improve the recanalization of blood vessels and restore the normal blood supply for brain tissue. However, thrombolytic therapy has strict requirements on the patient’s condition and treatment timing. Related research shows that timely successful reperfusion is the most effective treatment for patients with acute ischemic stroke.^6^ Therefore, EVT is more and more clinically preferred. Now relevant guidelines clearly state that: Recombinant tissue-type plasminogen activator (rt-PA) should be administered for intravenous thrombolysis for those within 4.5 hours since onset; for those who are within 4.5 hours since onset (time window) and also have anterior circulation large artery occlusion, the EVT based on the new generation of recyclable stent is recommended.^7^ However, revascularization of the blood vessel are no long considered for patients beyond the time window.

The concept of time window is certainly important, but for patients who are beyond the time window, timely revascularization and protection of the pathophysiological status of ischemic brain tissue may be more beneficial to patients.^8^ A prospective study assessed stroke progression through a time window of 6 to 16 hours, and studied stroke patients whose clinical images did not match and with time window of 6 to 24 hours. The results showed that thrombectomy in stroke patients could benefit them more.^9^

With the rapid development of neuroimaging technology, more accurate and comprehensive assessment of acute ischemic stroke using multi-modality of MRI has become a reality. Multi-mode MRI, including DWI, FLAIR, T1 and T2, has unique advantages in the judgment of the central location of infarction, the discovery of small lesions, chronic ischemic changes, the evaluation of collateral circulation, brain tissue ischemia, and reperfusion injury. At present, multi-mode MRI-guided reperfusion therapy mainly relies on two mismatch methods. One is to use penumbra theory to identify brain tissues at risk of ischemia, namely ischemic penumbra images (DWI/PWI mismatch).^10^ The other is the mismatch method of pathological and physiological images of acute cerebral ischemia, that is, DWI-FLAIR mismatch.^11^ In acute cerebral ischemia, cell edema occurs first, followed by angioedema. DWI is the most sensitive non-invasive method for detecting cytotoxic edema. It shows a high signal on DWI, and the signal abnormality in the infarct area can be found earlier than the ordinary MRI sequence.

Flair sequence is to weaken the signal of cerebrospinal fluid, reduce or eliminate the effect of volume-effect caused by cerebrospinal fluid, thereby improving the contrast between the lesion and normal tissues. Its high signal can reflect vasogenic edema caused by cerebral ischemia and hypoxia, indicating that the integrity of the blood-brain barrier is destroyed. It can also show signs of chronic cerebral ischemia, leukodystrophy, and old cerebral infarction. Therefore, the DWI-Flair mismatch reflects ischemia and hypoxia in the brain tissue, while vascular abnormalities and brain tissue necrosis have not yet formed, and the brain tissue can often be recovered after recanalization, which is often used clinically as an important imaging basis for judging the effect of thrombolysis beyond time window.^12^ We use it as the basis for EVT beyond the time window, and it has been confirmed that the symptom scores and the infarct volume before and after treatment under DWI-Flair mismatch have significant changes within 6 to 24 hours of onset.

The main complication after recanalization by EVT is intracranial hemorrhage, which is related to reperfusion injury after ischemia.^13^ In this study, the incidence of intracranial hemorrhage beyond time window is 12.5%. Although there are more cases than those within the time window (5:1), it is not statistically significant, suggesting that performing EVT is safe in patients beyond the time window under DWI-FLAIR mismatch. DWI-FLAIR mismatch can be used as an objective imaging basis for patients receiving EVT beyond time window. Mokin et al.^14^ believe that for patients with time window exceeding eight hour, successful reflow within three minutes is an independent predictor of good prognosis. Therefore, for such patients, there is still a need for active diagnosis and treatment, and to minimize the disability rate and increase the recovery rate.

### Limitations of the study

In this study, the sample size was small, and the follow-up time was short. More sample size and longer follow-up time are required to determine the long-term efficacy in patients receiving EVT beyond time window.

For ensuring patient safety and treatment effect, the time window is set as 6~24 hours. It is unclear whether patients receiving EVT beyond time window of more than 24 hours under DWI-FLAIR mismatch will have the same efficacy.In summary, for patients with ischemic stroke beyond the time window, DWI-FLAIR mismatch is as an objective imaging basis for EVT. If the patient’s indications are properly controlled, good therapeutic effects can still be achieved.

## CONCLUSION

For patients with intracranial hemorrhage that exceeds the time window, it is safe to receive EVT in the case of a DWI-FLAIR mismatch. DWI-FLAIR mismatch can be used as an objective imaging basis for intravascular interventional therapy in patients with stroke with over-time window and large vessel occlusion. It has the advantages of short examination time, non-invasiveness, no need for contrast agents, simple implementation, clear guidance. in addition, the long-term recovery effect of the patients within the time window will be better than that of patients beyond the time window.

### Authors’ Contributions:

**SC and** **HD** designed this study and prepared this manuscript, collected and analyzed clinical data, revised this manuscript, and they are responsible and accountable for the accuracy or integrity of the work.
